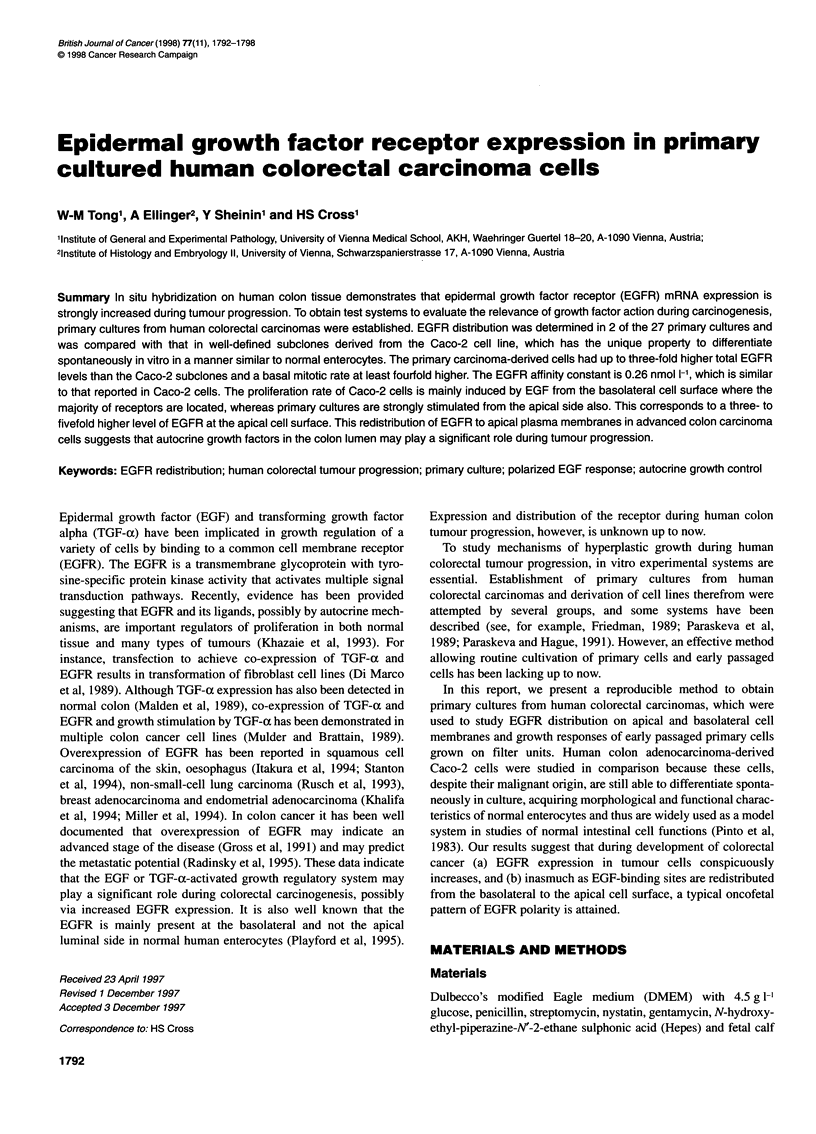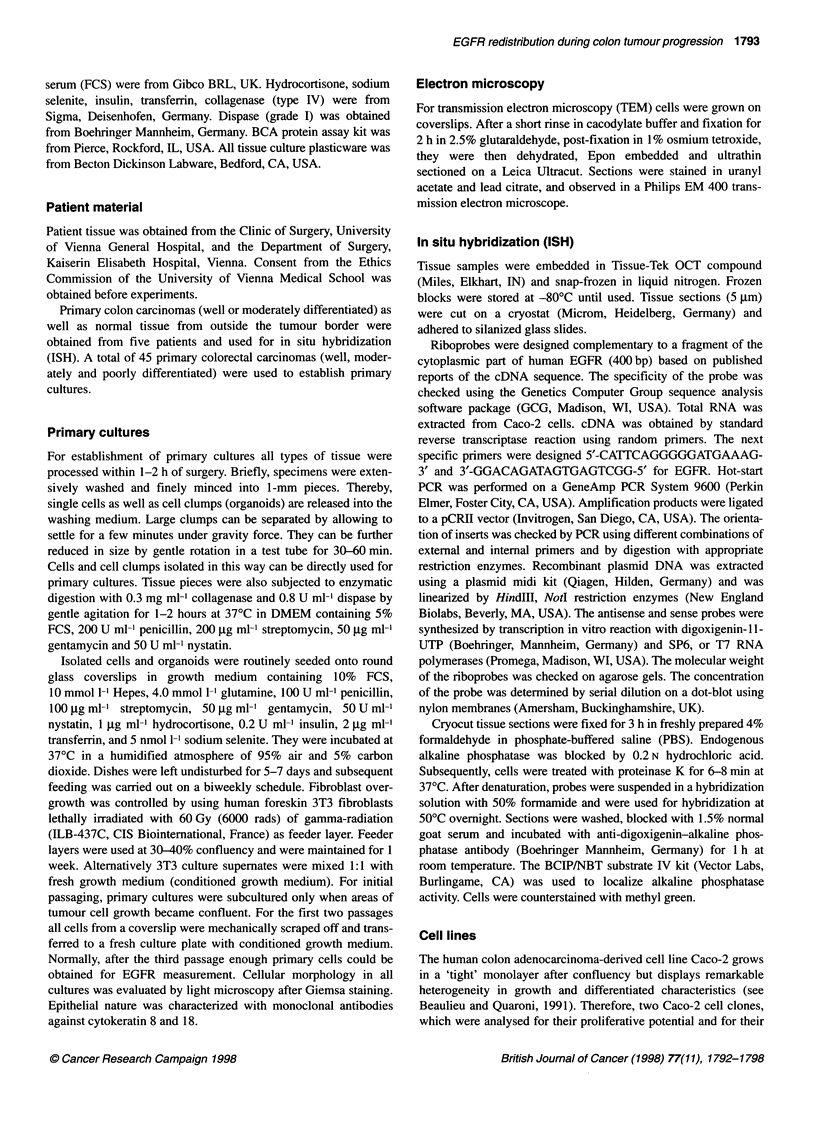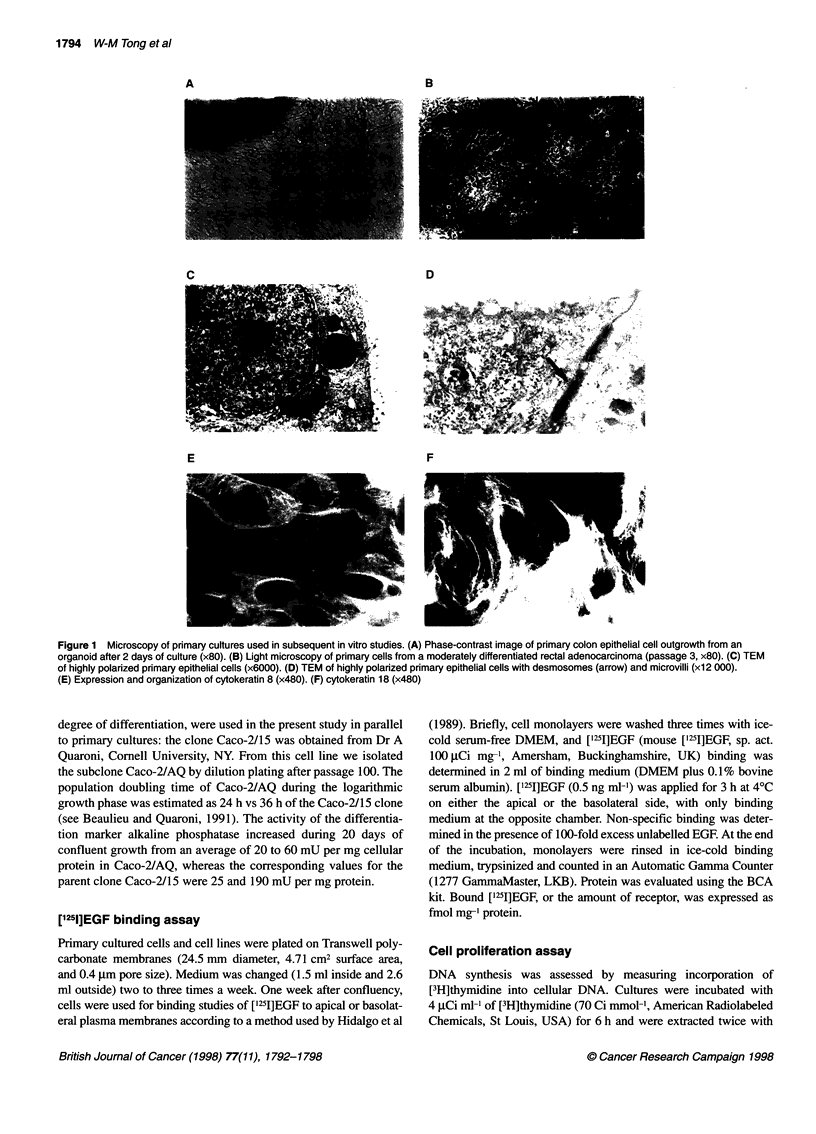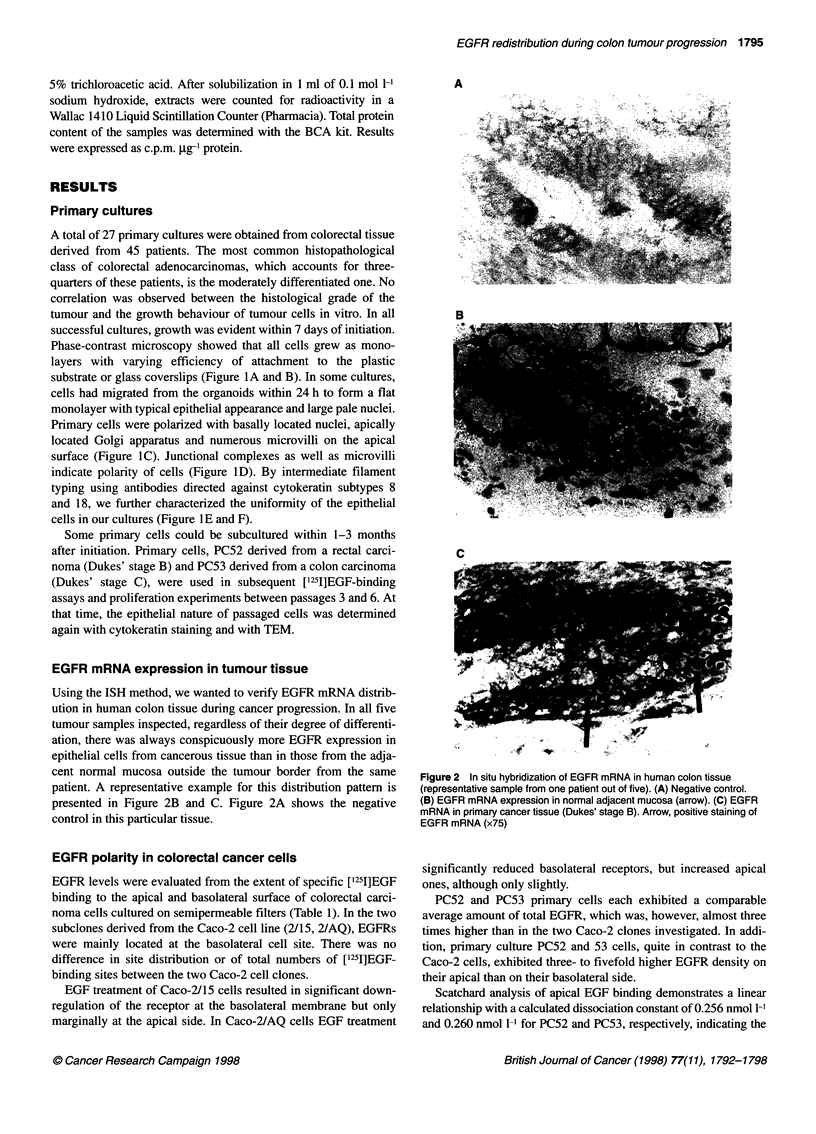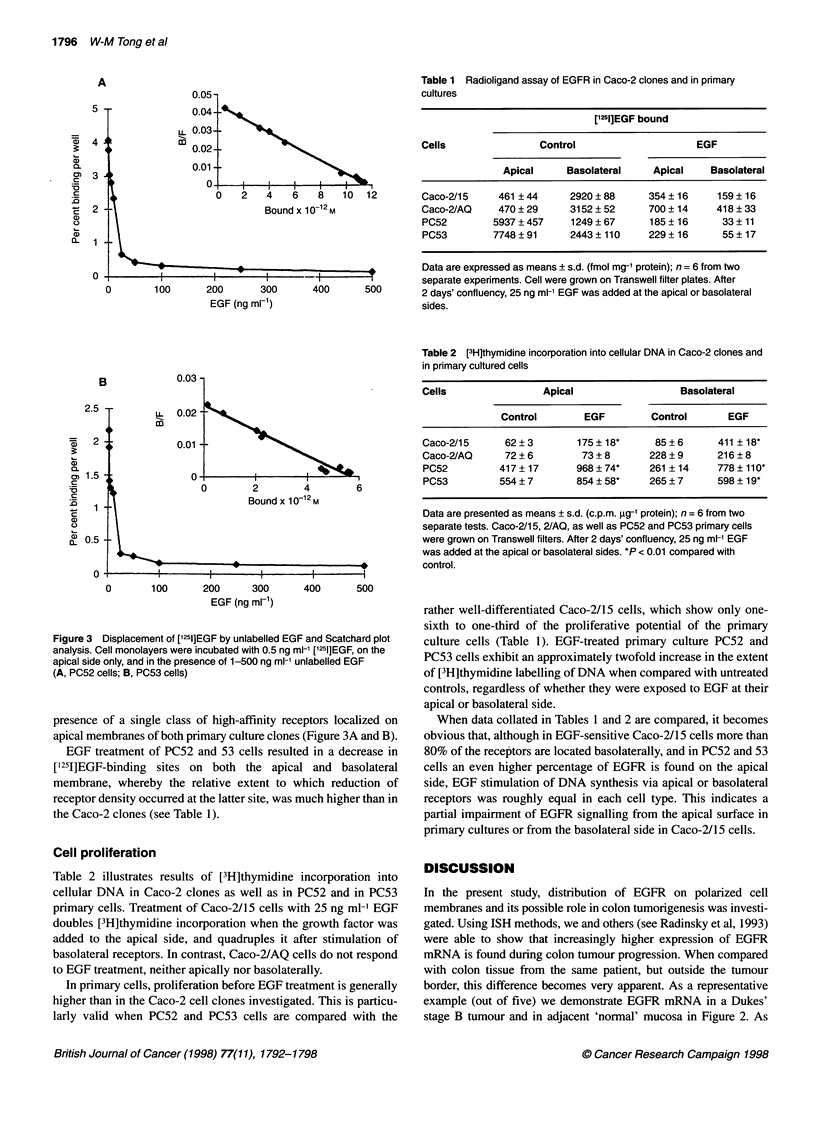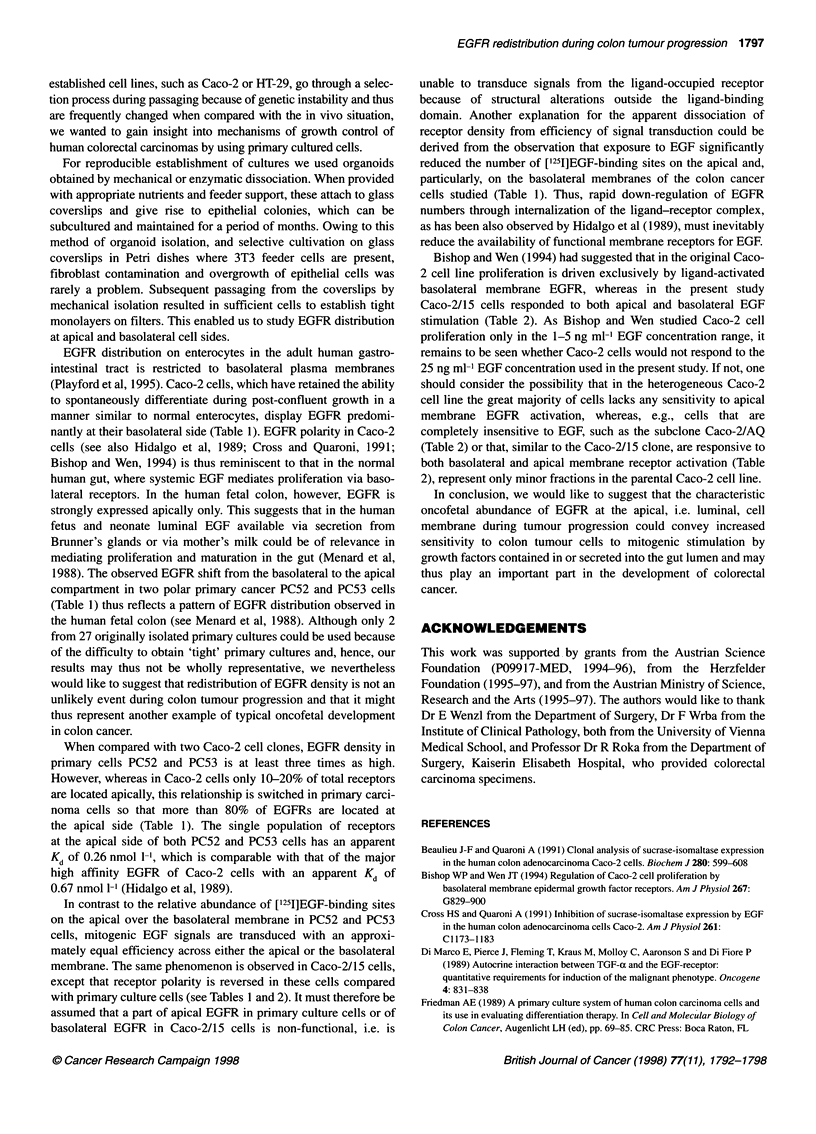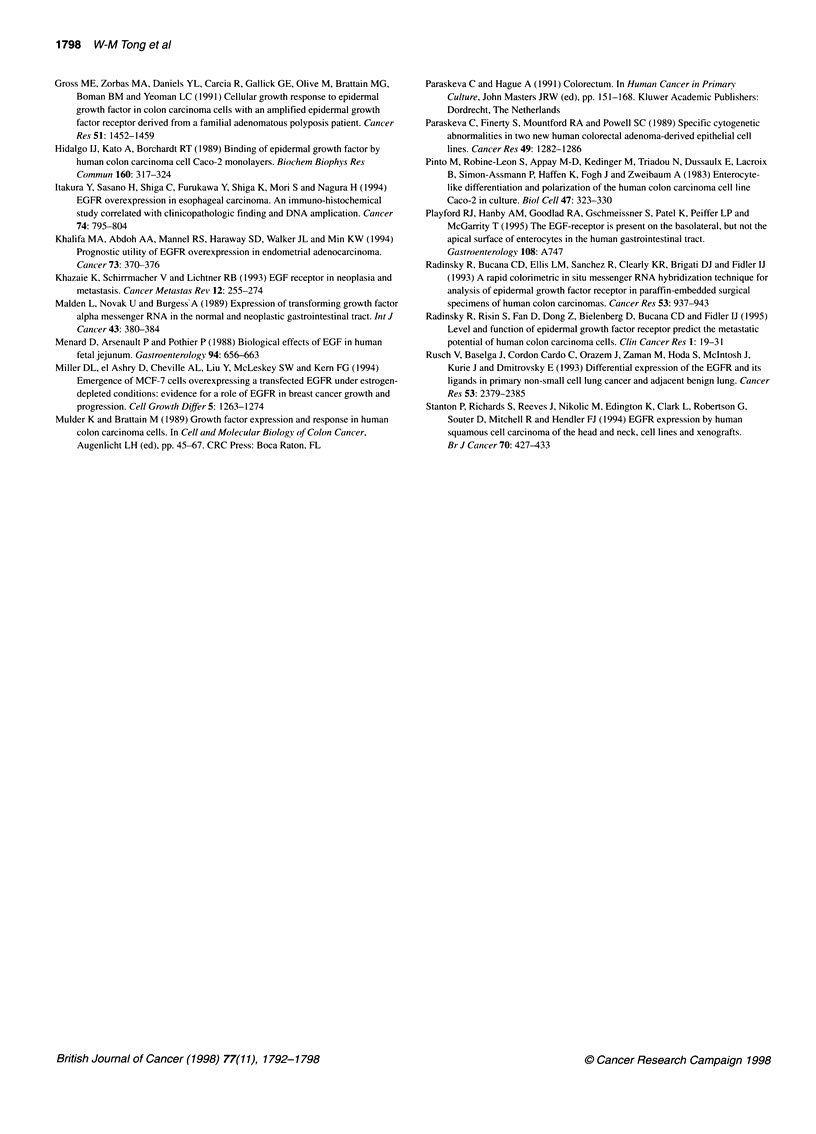# Epidermal growth factor receptor expression in primary cultured human colorectal carcinoma cells.

**DOI:** 10.1038/bjc.1998.298

**Published:** 1998-06

**Authors:** W. M. Tong, A. Ellinger, Y. Sheinin, H. S. Cross

**Affiliations:** Institute of General and Experimental Pathology, University of Vienna Medical School, Austria.

## Abstract

**Images:**


British Journal of Cancer (1998) 77(11), 1792–1798
© 1998 Cancer Research Campaign

### Epidermal growth factor receptor expression in primary cultured human colorectal carcinoma cells

**W-M Tong[1], A Ellinger[2], Y Sheinin[1] and HS Cross[1]**

[1]Institute of General and Experimental Pathology, University of Vienna Medical School, AKH, Waehringer Guertel 18–20, A-1090 Vienna, Austria;
[2]Institute of Histology and Embryology II, University of Vienna, Schwarzspanierstrasse 17, A-1090 Vienna, Austria

**Summary** In situ hybridization on human colon tissue demonstrates that epidermal growth factor receptor (EGFR) mRNA expression is strongly increased during tumour progression. To obtain test systems to evaluate the relevance of growth factor action during carcinogenesis, primary cultures from human colorectal carcinomas were established. EGFR distribution was determined in 2 of the 27 primary cultures and was compared with that in well-defined subclones derived from the Caco-2 cell line, which has the unique property to differentiate spontaneously in vitro in a manner similar to normal enterocytes. The primary carcinoma-derived cells had up to three-fold higher total EGFR levels than the Caco-2 subclones and a basal mitotic rate at least fourfold higher. The EGFR affinity constant is 0.26 nmol $l^{-1}$, which is similar to that reported in Caco-2 cells. The proliferation rate of Caco-2 cells is mainly induced by EGF from the basolateral cell surface where the majority of receptors are located, whereas primary cultures are strongly stimulated from the apical side also. This corresponds to a three- to fivefold higher level of EGFR at the apical cell surface. This redistribution of EGFR to apical plasma membranes in advanced colon carcinoma cells suggests that autocrine growth factors in the colon lumen may play a significant role during tumour progression.

**Keywords:** EGFR redistribution; human colorectal tumour progression; primary culture; polarized EGF response; autocrine growth control

Epidermal growth factor (EGF) and transforming growth factor alpha (TGF-α) have been implicated in growth regulation of a variety of cells by binding to a common cell membrane receptor (EGFR). The EGFR is a transmembrane glycoprotein with tyrosine-specific protein kinase activity that activates multiple signal transduction pathways. Recently, evidence has been provided suggesting that EGFR and its ligands, possibly by autocrine mechanisms, are important regulators of proliferation in both normal tissue and many types of tumours (Khazaie et al, 1993). For instance, transfection to achieve co-expression of TGF-α and EGFR results in transformation of fibroblast cell lines (Di Marco et al, 1989). Although TGF-α expression has also been detected in normal colon (Malden et al, 1989), co-expression of TGF-α and EGFR and growth stimulation by TGF-α has been demonstrated in multiple colon cancer cell lines (Mulder and Brattain, 1989). Overexpression of EGFR has been reported in squamous cell carcinoma of the skin, oesophagus (Itakura et al, 1994; Stanton et al, 1994), non-small-cell lung carcinoma (Rusch et al, 1993), breast adenocarcinoma and endometrial adenocarcinoma (Khalifa et al, 1994; Miller et al, 1994). In colon cancer it has been well documented that overexpression of EGFR may indicate an advanced stage of the disease (Gross et al, 1991) and may predict the metastatic potential (Radinsky et al, 1995). These data indicate that the EGF or TGF-α-activated growth regulatory system may play a significant role during colorectal carcinogenesis, possibly via increased EGFR expression. It is also well known that the EGFR is mainly present at the basolateral and not the apical luminal side in normal human enterocytes (Playford et al, 1995).

*Received 23 April 1997*
*Revised 1 December 1997*
*Accepted 3 December 1997*

*Correspondence to:* HS Cross

Expression and distribution of the receptor during human colon tumour progression, however, is unknown up to now.

To study mechanisms of hyperplastic growth during human colorectal tumour progression, in vitro experimental systems are essential. Establishment of primary cultures from human colorectal carcinomas and derivation of cell lines therefrom were attempted by several groups, and some systems have been described (see, for example, Friedman, 1989; Paraskeva et al, 1989; Paraskeva and Hague, 1991). However, an effective method allowing routine cultivation of primary cells and early passaged cells has been lacking up to now.

In this report, we present a reproducible method to obtain primary cultures from human colorectal carcinomas, which were used to study EGFR distribution on apical and basolateral cell membranes and growth responses of early passaged primary cells grown on filter units. Human colon adenocarcinoma-derived Caco-2 cells were studied in comparison because these cells, despite their malignant origin, are still able to differentiate spontaneously in culture, acquiring morphological and functional characteristics of normal enterocytes and thus are widely used as a model system in studies of normal intestinal cell functions (Pinto et al, 1983). Our results suggest that during development of colorectal cancer (a) EGFR expression in tumour cells conspicuously increases, and (b) inasmuch as EGF-binding sites are redistributed from the basolateral to the apical cell surface, a typical oncofetal pattern of EGFR polarity is attained.

### MATERIALS AND METHODS

### Materials

Dulbecco's modified Eagle medium (DMEM) with 4.5 g $l^{-1}$ glucose, penicillin, streptomycin, nystatin, gentamycin, *N*-hydroxyethyl-piperazine-*N*′-2-ethane sulphonic acid (Hepes) and fetal calf serum (FCS) were from Gibco BRL, UK. Hydrocortisone, sodium selenite, insulin, transferrin, collagenase (type IV) were from Sigma, Deisenhofen, Germany. Dispase (grade I) was obtained from Boehringer Mannheim, Germany. BCA protein assay kit was from Pierce, Rockford, IL, USA. All tissue culture plasticware was from Becton Dickinson Labware, Bedford, CA, USA.

### Patient material

Patient tissue was obtained from the Clinic of Surgery, University of Vienna General Hospital, and the Department of Surgery, Kaiserin Elisabeth Hospital, Vienna. Consent from the Ethics Commission of the University of Vienna Medical School was obtained before experiments.

Primary colon carcinomas (well or moderately differentiated) as well as normal tissue from outside the tumour border were obtained from five patients and used for in situ hybridization (ISH). A total of 45 primary colorectal carcinomas (well, moderately and poorly differentiated) were used to establish primary cultures.

### Primary cultures

For establishment of primary cultures all types of tissue were processed within 1–2 h of surgery. Briefly, specimens were extensively washed and finely minced into 1-mm pieces. Thereby, single cells as well as cell clumps (organoids) are released into the washing medium. Large clumps can be separated by allowing to settle for a few minutes under gravity force. They can be further reduced in size by gentle rotation in a test tube for 30–60 min. Cells and cell clumps isolated in this way can be directly used for primary cultures. Tissue pieces were also subjected to enzymatic digestion with 0.3 mg $ml^{-1}$ collagenase and 0.8 U $ml^{-1}$ dispase by gentle agitation for 1–2 hours at 37°C in DMEM containing 5% FCS, 200 U $ml^{-1}$ penicillin, 200 µg $ml^{-1}$ streptomycin, 50 µg $ml^{-1}$ gentamycin and 50 U $ml^{-1}$ nystatin.

Isolated cells and organoids were routinely seeded onto round glass coverslips in growth medium containing 10% FCS, 10 mmol $l^{-1}$ Hepes, 4.0 mmol $l^{-1}$ glutamine, 100 U $ml^{-1}$ penicillin, 100 µg $ml^{-1}$ streptomycin, 50 µg $ml^{-1}$ gentamycin, 50 U $ml^{-1}$ nystatin, 1 µg $ml^{-1}$ hydrocortisone, 0.2 U $ml^{-1}$ insulin, 2 µg $ml^{-1}$ transferrin, and 5 nmol $l^{-1}$ sodium selenite. They were incubated at 37°C in a humidified atmosphere of 95% air and 5% carbon dioxide. Dishes were left undisturbed for 5–7 days and subsequent feeding was carried out on a biweekly schedule. Fibroblast overgrowth was controlled by using human foreskin 3T3 fibroblasts lethally irradiated with 60 Gy (6000 rads) of gamma-radiation (ILB-437C, CIS Biointernational, France) as feeder layer. Feeder layers were used at 30–40% confluency and were maintained for 1 week. Alternatively 3T3 culture supernates were mixed 1:1 with fresh growth medium (conditioned growth medium). For initial passaging, primary cultures were subcultured only when areas of tumour cell growth became confluent. For the first two passages all cells from a coverslip were mechanically scraped off and transferred to a fresh culture plate with conditioned growth medium. Normally, after the third passage enough primary cells could be obtained for EGFR measurement. Cellular morphology in all cultures was evaluated by light microscopy after Giemsa staining. Epithelial nature was characterized with monoclonal antibodies against cytokeratin 8 and 18.

### Electron microscopy

For transmission electron microscopy (TEM) cells were grown on coverslips. After a short rinse in cacodylate buffer and fixation for 2 h in 2.5% glutaraldehyde, post-fixation in 1% osmium tetroxide, they were then dehydrated, Epon embedded and ultrathin sectioned on a Leica Ultracut. Sections were stained in uranyl acetate and lead citrate, and observed in a Philips EM 400 transmission electron microscope.

### In situ hybridization (ISH)

Tissue samples were embedded in Tissue-Tek OCT compound (Miles, Elkhart, IN) and snap-frozen in liquid nitrogen. Frozen blocks were stored at –80°C until used. Tissue sections (5 µm) were cut on a cryostat (Microm, Heidelberg, Germany) and adhered to silanized glass slides.

Riboprobes were designed complementary to a fragment of the cytoplasmic part of human EGFR (400 bp) based on published reports of the cDNA sequence. The specificity of the probe was checked using the Genetics Computer Group sequence analysis software package (GCG, Madison, WI, USA). Total RNA was extracted from Caco-2 cells. cDNA was obtained by standard reverse transcriptase reaction using random primers. The next specific primers were designed 5′-CATTCAGGGGGATGAAAG-3′ and 3′-GGACAGATAGTGAGTCGG-5′ for EGFR. Hot-start PCR was performed on a GeneAmp PCR System 9600 (Perkin Elmer, Foster City, CA, USA). Amplification products were ligated to a pCRII vector (Invitrogen, San Diego, CA, USA). The orientation of inserts was checked by PCR using different combinations of external and internal primers and by digestion with appropriate restriction enzymes. Recombinant plasmid DNA was extracted using a plasmid midi kit (Qiagen, Hilden, Germany) and was linearized by *Hin*dIII, *Not*I restriction enzymes (New England Biolabs, Beverly, MA, USA). The antisense and sense probes were synthesized by transcription in vitro reaction with digoxigenin-11-UTP (Boehringer, Mannheim, Germany) and SP6, or T7 RNA polymerases (Promega, Madison, WI, USA). The molecular weight of the riboprobes was checked on agarose gels. The concentration of the probe was determined by serial dilution on a dot-blot using nylon membranes (Amersham, Buckinghamshire, UK).

Cryocut tissue sections were fixed for 3 h in freshly prepared 4% formaldehyde in phosphate-buffered saline (PBS). Endogenous alkaline phosphatase was blocked by 0.2 N hydrochloric acid. Subsequently, cells were treated with proteinase K for 6–8 min at 37°C. After denaturation, probes were suspended in a hybridization solution with 50% formamide and were used for hybridization at 50°C overnight. Sections were washed, blocked with 1.5% normal goat serum and incubated with anti-digoxigenin–alkaline phosphatase antibody (Boehringer Mannheim, Germany) for 1 h at room temperature. The BCIP/NBT substrate IV kit (Vector Labs, Burlingame, CA) was used to localize alkaline phosphatase activity. Cells were counterstained with methyl green.

### Cell lines

The human colon adenocarcinoma-derived cell line Caco-2 grows in a 'tight' monolayer after confluency but displays remarkable heterogeneity in growth and differentiated characteristics (see Beaulieu and Quaroni, 1991). Therefore, two Caco-2 cell clones, which were analysed for their proliferative potential and for their

© Cancer Research Campaign 1998

**Figure 1** Microscopy of primary cultures used in subsequent in vitro studies. (**A**) Phase-contrast image of primary colon epithelial cell outgrowth from an organoid after 2 days of culture (×80). (**B**) Light microscopy of primary cells from a moderately differentiated rectal adenocarcinoma (passage 3, ×80). (**C**) TEM of highly polarized primary epithelial cells (×6000). (**D**) TEM of highly polarized primary epithelial cells with desmosomes (arrow) and microvilli (×12 000). (**E**) Expression and organization of cytokeratin 8 (×480). (**F**) cytokeratin 18 (×480)

degree of differentiation, were used in the present study in parallel to primary cultures: the clone Caco-2/15 was obtained from Dr A Quaroni, Cornell University, NY. From this cell line we isolated the subclone Caco-2/AQ by dilution plating after passage 100. The population doubling time of Caco-2/AQ during the logarithmic growth phase was estimated as 24 h vs 36 h of the Caco-2/15 clone (see Beaulieu and Quaroni, 1991). The activity of the differentiation marker alkaline phosphatase increased during 20 days of confluent growth from an average of 20 to 60 mU per mg cellular protein in Caco-2/AQ, whereas the corresponding values for the parent clone Caco-2/15 were 25 and 190 mU per mg protein.

### [$^{125}$I]EGF binding assay

Primary cultured cells and cell lines were plated on Transwell polycarbonate membranes (24.5 mm diameter, 4.71 cm$^2$ surface area, and 0.4 μm pore size). Medium was changed (1.5 ml inside and 2.6 ml outside) two to three times a week. One week after confluency, cells were used for binding studies of [$^{125}$I]EGF to apical or basolateral plasma membranes according to a method used by Hidalgo et al (1989). Briefly, cell monolayers were washed three times with ice-cold serum-free DMEM, and [$^{125}$I]EGF (mouse [$^{125}$I]EGF, sp. act. 100 μCi mg$^{-1}$, Amersham, Buckinghamshire, UK) binding was determined in 2 ml of binding medium (DMEM plus 0.1% bovine serum albumin). [$^{125}$I]EGF (0.5 ng ml$^{-1}$) was applied for 3 h at 4°C on either the apical or the basolateral side, with only binding medium at the opposite chamber. Non-specific binding was determined in the presence of 100-fold excess unlabelled EGF. At the end of the incubation, monolayers were rinsed in ice-cold binding medium, trypsinized and counted in an Automatic Gamma Counter (1277 GammaMaster, LKB). Protein was evaluated using the BCA kit. Bound [$^{125}$I]EGF, or the amount of receptor, was expressed as fmol mg$^{-1}$ protein.

### Cell proliferation assay

DNA synthesis was assessed by measuring incorporation of [$^3$H]thymidine into cellular DNA. Cultures were incubated with 4 μCi ml$^{-1}$ of [$^3$H]thymidine (70 Ci mmol$^{-1}$, American Radiolabeled Chemicals, St Louis, USA) for 6 h and were extracted twice with

© Cancer Research Campaign 1998

5% trichloroacetic acid. After solubilization in 1 ml of 0.1 mol $l^{-1}$ sodium hydroxide, extracts were counted for radioactivity in a Wallac 1410 Liquid Scintillation Counter (Pharmacia). Total protein content of the samples was determined with the BCA kit. Results were expressed as c.p.m. $\mu g^{-1}$ protein.

### RESULTS

### Primary cultures

A total of 27 primary cultures were obtained from colorectal tissue derived from 45 patients. The most common histopathological class of colorectal adenocarcinomas, which accounts for three-quarters of these patients, is the moderately differentiated one. No correlation was observed between the histological grade of the tumour and the growth behaviour of tumour cells in vitro. In all successful cultures, growth was evident within 7 days of initiation. Phase-contrast microscopy showed that all cells grew as monolayers with varying efficiency of attachment to the plastic substrate or glass coverslips (Figure 1A and B). In some cultures, cells had migrated from the organoids within 24 h to form a flat monolayer with typical epithelial appearance and large pale nuclei. Primary cells were polarized with basally located nuclei, apically located Golgi apparatus and numerous microvilli on the apical surface (Figure 1C). Junctional complexes as well as microvilli indicate polarity of cells (Figure 1D). By intermediate filament typing using antibodies directed against cytokeratin subtypes 8 and 18, we further characterized the uniformity of the epithelial cells in our cultures (Figure 1E and F).

Some primary cells could be subcultured within 1–3 months after initiation. Primary cells, PC52 derived from a rectal carcinoma (Dukes' stage B) and PC53 derived from a colon carcinoma (Dukes' stage C), were used in subsequent [$^{125}$I]EGF-binding assays and proliferation experiments between passages 3 and 6. At that time, the epithelial nature of passaged cells was determined again with cytokeratin staining and with TEM.

### EGFR mRNA expression in tumour tissue

Using the ISH method, we wanted to verify EGFR mRNA distribution in human colon tissue during cancer progression. In all five tumour samples inspected, regardless of their degree of differentiation, there was always conspicuously more EGFR expression in epithelial cells from cancerous tissue than in those from the adjacent normal mucosa outside the tumour border from the same patient. A representative example for this distribution pattern is presented in Figure 2B and C. Figure 2A shows the negative control in this particular tissue.

**Figure 2** In situ hybridization of EGFR mRNA in human colon tissue (representative sample from one patient out of five). (**A**) Negative control. (**B**) EGFR mRNA expression in normal adjacent mucosa (arrow). (**C**) EGFR mRNA in primary cancer tissue (Dukes' stage B). Arrow, positive staining of EGFR mRNA (×75)

### EGFR polarity in colorectal cancer cells

EGFR levels were evaluated from the extent of specific [$^{125}$I]EGF binding to the apical and basolateral surface of colorectal carcinoma cells cultured on semipermeable filters (Table 1). In the two subclones derived from the Caco-2 cell line (2/15, 2/AQ), EGFRs were mainly located at the basolateral cell site. There was no difference in site distribution or of total numbers of [$^{125}$I]EGF-binding sites between the two Caco-2 cell clones.

EGF treatment of Caco-2/15 cells resulted in significant down-regulation of the receptor at the basolateral membrane but only marginally at the apical side. In Caco-2/AQ cells EGF treatment significantly reduced basolateral receptors, but increased apical ones, although only slightly.

PC52 and PC53 primary cells each exhibited a comparable average amount of total EGFR, which was, however, almost three times higher than in the two Caco-2 clones investigated. In addition, primary culture PC52 and 53 cells, quite in contrast to the Caco-2 cells, exhibited three- to fivefold higher EGFR density on their apical than on their basolateral side.

Scatchard analysis of apical EGF binding demonstrates a linear relationship with a calculated dissociation constant of 0.256 nmol $l^{-1}$ and 0.260 nmol $l^{-1}$ for PC52 and PC53, respectively, indicating the

**Figure 3** Displacement of $[^{125}I]$EGF by unlabelled EGF and Scatchard plot analysis. Cell monolayers were incubated with 0.5 ng $ml^{-1}$ $[^{125}I]$EGF, on the apical side only, and in the presence of 1–500 ng $ml^{-1}$ unlabelled EGF (**A**, PC52 cells; **B**, PC53 cells)

presence of a single class of high-affinity receptors localized on apical membranes of both primary culture clones (Figure 3A and B).

EGF treatment of PC52 and 53 cells resulted in a decrease in $[^{125}I]$EGF-binding sites on both the apical and basolateral membrane, whereby the relative extent to which reduction of receptor density occurred at the latter site, was much higher than in the Caco-2 clones (see Table 1).

### Cell proliferation

Table 2 illustrates results of $[^3H]$thymidine incorporation into cellular DNA in Caco-2 clones as well as in PC52 and in PC53 primary cells. Treatment of Caco-2/15 cells with 25 ng $ml^{-1}$ EGF doubles $[^3H]$thymidine incorporation when the growth factor was added to the apical side, and quadruples it after stimulation of basolateral receptors. In contrast, Caco-2/AQ cells do not respond to EGF treatment, neither apically nor basolaterally.

In primary cells, proliferation before EGF treatment is generally higher than in the Caco-2 cell clones investigated. This is particularly valid when PC52 and PC53 cells are compared with the rather well-differentiated Caco-2/15 cells, which show only one-sixth to one-third of the proliferative potential of the primary culture cells (Table 1). EGF-treated primary culture PC52 and PC53 cells exhibit an approximately twofold increase in the extent of $[^3H]$thymidine labelling of DNA when compared with untreated controls, regardless of whether they were exposed to EGF at their apical or basolateral side.

When data collated in Tables 1 and 2 are compared, it becomes obvious that, although in EGF-sensitive Caco-2/15 cells more than 80% of the receptors are located basolaterally, and in PC52 and 53 cells an even higher percentage of EGFR is found on the apical side, EGF stimulation of DNA synthesis via apical or basolateral receptors was roughly equal in each cell type. This indicates a partial impairment of EGFR signalling from the apical surface in primary cultures or from the basolateral side in Caco-2/15 cells.

**Table 1** Radioligand assay of EGFR in Caco-2 clones and in primary cultures

| | $[^{125}I]$EGF bound | | | |
|---|---|---|---|---|
| **Cells** | **Control** | | **EGF** | |
| | **Apical** | **Basolateral** | **Apical** | **Basolateral** |
| Caco-2/15 | 461 ± 44 | 2920 ± 88 | 354 ± 16 | 159 ± 16 |
| Caco-2/AQ | 470 ± 29 | 3152 ± 52 | 700 ± 14 | 418 ± 33 |
| PC52 | 5937 ± 457 | 1249 ± 67 | 185 ± 16 | 33 ± 11 |
| PC53 | 7748 ± 91 | 2443 ± 110 | 229 ± 16 | 55 ± 17 |

Data are expressed as means ± s.d. (fmol $mg^{-1}$ protein); $n = 6$ from two separate experiments. Cell were grown on Transwell filter plates. After 2 days' confluency, 25 ng $ml^{-1}$ EGF was added at the apical or basolateral sides.

**Table 2** $[^3H]$thymidine incorporation into cellular DNA in Caco-2 clones and in primary cultured cells

| **Cells** | **Apical** | | **Basolateral** | |
|---|---|---|---|---|
| | **Control** | **EGF** | **Control** | **EGF** |
| Caco-2/15 | 62 ± 3 | 175 ± 18* | 85 ± 6 | 411 ± 18* |
| Caco-2/AQ | 72 ± 6 | 73 ± 8 | 228 ± 9 | 216 ± 8 |
| PC52 | 417 ± 17 | 968 ± 74* | 261 ± 14 | 778 ± 110* |
| PC53 | 554 ± 7 | 854 ± 58* | 265 ± 7 | 598 ± 19* |

Data are presented as means ± s.d. (c.p.m. $\mu g^{-1}$ protein); $n = 6$ from two separate tests. Caco-2/15, 2/AQ, as well as PC52 and PC53 primary cells were grown on Transwell filters. After 2 days' confluency, 25 ng $ml^{-1}$ EGF was added at the apical or basolateral sides. *$P < 0.01$ compared with control.

### DISCUSSION

In the present study, distribution of EGFR on polarized cell membranes and its possible role in colon tumorigenesis was investigated. Using ISH methods, we and others (see Radinsky et al, 1993) were able to show that increasingly higher expression of EGFR mRNA is found during colon tumour progression. When compared with colon tissue from the same patient, but outside the tumour border, this difference becomes very apparent. As a representative example (out of five) we demonstrate EGFR mRNA in a Dukes' stage B tumour and in adjacent 'normal' mucosa in Figure 2. As

© Cancer Research Campaign 1998

established cell lines, such as Caco-2 or HT-29, go through a selection process during passaging because of genetic instability and thus are frequently changed when compared with the in vivo situation, we wanted to gain insight into mechanisms of growth control of human colorectal carcinomas by using primary cultured cells.

For reproducible establishment of cultures we used organoids obtained by mechanical or enzymatic dissociation. When provided with appropriate nutrients and feeder support, these attach to glass coverslips and give rise to epithelial colonies, which can be subcultured and maintained for a period of months. Owing to this method of organoid isolation, and selective cultivation on glass coverslips in Petri dishes where 3T3 feeder cells are present, fibroblast contamination and overgrowth of epithelial cells was rarely a problem. Subsequent passaging from the coverslips by mechanical isolation resulted in sufficient cells to establish tight monolayers on filters. This enabled us to study EGFR distribution at apical and basolateral cell sides.

EGFR distribution on enterocytes in the adult human gastrointestinal tract is restricted to basolateral plasma membranes (Playford et al, 1995). Caco-2 cells, which have retained the ability to spontaneously differentiate during post-confluent growth in a manner similar to normal enterocytes, display EGFR predominantly at their basolateral side (Table 1). EGFR polarity in Caco-2 cells (see also Hidalgo et al, 1989; Cross and Quaroni, 1991; Bishop and Wen, 1994) is thus reminiscent to that in the normal human gut, where systemic EGF mediates proliferation via basolateral receptors. In the human fetal colon, however, EGFR is strongly expressed apically only. This suggests that in the human fetus and neonate luminal EGF available via secretion from Brunner's glands or via mother's milk could be of relevance in mediating proliferation and maturation in the gut (Menard et al, 1988). The observed EGFR shift from the basolateral to the apical compartment in two polar primary cancer PC52 and PC53 cells (Table 1) thus reflects a pattern of EGFR distribution observed in the human fetal colon (see Menard et al, 1988). Although only 2 from 27 originally isolated primary cultures could be used because of the difficulty to obtain 'tight' primary cultures and, hence, our results may thus not be wholly representative, we nevertheless would like to suggest that redistribution of EGFR density is not an unlikely event during colon tumour progression and that it might thus represent another example of typical oncofetal development in colon cancer.

When compared with two Caco-2 cell clones, EGFR density in primary cells PC52 and PC53 is at least three times as high. However, whereas in Caco-2 cells only 10–20% of total receptors are located apically, this relationship is switched in primary carcinoma cells so that more than 80% of EGFRs are located at the apical side (Table 1). The single population of receptors at the apical side of both PC52 and PC53 cells has an apparent $K_d$ of 0.26 nmol $l^{-1}$, which is comparable with that of the major high affinity EGFR of Caco-2 cells with an apparent $K_d$ of 0.67 nmol $l^{-1}$ (Hidalgo et al, 1989).

In contrast to the relative abundance of [$^{125}$I]EGF-binding sites on the apical over the basolateral membrane in PC52 and PC53 cells, mitogenic EGF signals are transduced with an approximately equal efficiency across either the apical or the basolateral membrane. The same phenomenon is observed in Caco-2/15 cells, except that receptor polarity is reversed in these cells compared with primary culture cells (see Tables 1 and 2). It must therefore be assumed that a part of apical EGFR in primary culture cells or of basolateral EGFR in Caco-2/15 cells is non-functional, i.e. is unable to transduce signals from the ligand-occupied receptor because of structural alterations outside the ligand-binding domain. Another explanation for the apparent dissociation of receptor density from efficiency of signal transduction could be derived from the observation that exposure to EGF significantly reduced the number of [$^{125}$I]EGF-binding sites on the apical and, particularly, on the basolateral membranes of the colon cancer cells studied (Table 1). Thus, rapid down-regulation of EGFR numbers through internalization of the ligand–receptor complex, as has been also observed by Hidalgo et al (1989), must inevitably reduce the availability of functional membrane receptors for EGF.

Bishop and Wen (1994) had suggested that in the original Caco-2 cell line proliferation is driven exclusively by ligand-activated basolateral membrane EGFR, whereas in the present study Caco-2/15 cells responded to both apical and basolateral EGF stimulation (Table 2). As Bishop and Wen studied Caco-2 cell proliferation only in the 1–5 ng $ml^{-1}$ EGF concentration range, it remains to be seen whether Caco-2 cells would not respond to the 25 ng $ml^{-1}$ EGF concentration used in the present study. If not, one should consider the possibility that in the heterogeneous Caco-2 cell line the great majority of cells lacks any sensitivity to apical membrane EGFR activation, whereas, e.g., cells that are completely insensitive to EGF, such as the subclone Caco-2/AQ (Table 2) or that, similar to the Caco-2/15 clone, are responsive to both basolateral and apical membrane receptor activation (Table 2), represent only minor fractions in the parental Caco-2 cell line.

In conclusion, we would like to suggest that the characteristic oncofetal abundance of EGFR at the apical, i.e. luminal, cell membrane during tumour progression could convey increased sensitivity to colon tumour cells to mitogenic stimulation by growth factors contained in or secreted into the gut lumen and may thus play an important part in the development of colorectal cancer.

### ACKNOWLEDGEMENTS

This work was supported by grants from the Austrian Science Foundation (P09917-MED, 1994–96), from the Herzfelder Foundation (1995–97), and from the Austrian Ministry of Science, Research and the Arts (1995–97). The authors would like to thank Dr E Wenzl from the Department of Surgery, Dr F Wrba from the Institute of Clinical Pathology, both from the University of Vienna Medical School, and Professor Dr R Roka from the Department of Surgery, Kaiserin Elisabeth Hospital, who provided colorectal carcinoma specimens.

### REFERENCES

Beaulieu J-F and Quaroni A (1991) Clonal analysis of sucrase-isomaltase expression in the human colon adenocarcinoma Caco-2 cells. *Biochem J* **280**: 599–608

Bishop WP and Wen JT (1994) Regulation of Caco-2 cell proliferation by basolateral membrane epidermal growth factor receptors. *Am J Physiol* **267**: G829–900

Cross HS and Quaroni A (1991) Inhibition of sucrase-isomaltase expression by EGF in the human colon adenocarcinoma cells Caco-2. *Am J Physiol* **261**: C1173–1183

Di Marco E, Pierce J, Fleming T, Kraus M, Molloy C, Aaronson S and Di Fiore P (1989) Autocrine interaction between TGF-α and the EGF-receptor: quantitative requirements for induction of the malignant phenotype. *Oncogene* **4**: 831–838

Friedman AE (1989) A primary culture system of human colon carcinoma cells and its use in evaluating differentiation therapy. In *Cell and Molecular Biology of Colon Cancer*, Augenlicht LH (ed), pp. 69–85. CRC Press: Boca Raton, FL

© Cancer Research Campaign 1998 

Gross ME, Zorbas MA, Daniels YL, Carcia R, Gallick GE, Olive M, Brattain MG, Boman BM and Yeoman LC (1991) Cellular growth response to epidermal growth factor in colon carcinoma cells with an amplified epidermal growth factor receptor derived from a familial adenomatous polyposis patient. *Cancer Res* **51**: 1452–1459

Hidalgo IJ, Kato A, Borchardt RT (1989) Binding of epidermal growth factor by human colon carcinoma cell Caco-2 monolayers. *Biochem Biophys Res Commun* **160**: 317–324

Itakura Y, Sasano H, Shiga C, Furukawa Y, Shiga K, Mori S and Nagura H (1994) EGFR overexpression in esophageal carcinoma. An immuno-histochemical study correlated with clinicopathologic finding and DNA amplication. *Cancer* **74**: 795–804

Khalifa MA, Abdoh AA, Mannel RS, Haraway SD, Walker JL and Min KW (1994) Prognostic utility of EGFR overexpression in endometrial adenocarcinoma. *Cancer* **73**: 370–376

Khazaie K, Schirrmacher V and Lichtner RB (1993) EGF receptor in neoplasia and metastasis. *Cancer Metastas Rev* **12**: 255–274

Malden L, Novak U and Burgess A (1989) Expression of transforming growth factor alpha messenger RNA in the normal and neoplastic gastrointestinal tract. *Int J Cancer* **43**: 380–384

Menard D, Arsenault P and Pothier P (1988) Biological effects of EGF in human fetal jejunum. *Gastroenterology* **94**: 656–663

Miller DL, el Ashry D, Cheville AL, Liu Y, McLeskey SW and Kern FG (1994) Emergence of MCF-7 cells overexpressing a transfected EGFR under estrogen-depleted conditions: evidence for a role of EGFR in breast cancer growth and progression. *Cell Growth Differ* **5**: 1263–1274

Mulder K and Brattain M (1989) Growth factor expression and response in human colon carcinoma cells. In *Cell and Molecular Biology of Colon Cancer*, Augenlicht LH (ed), pp. 45–67. CRC Press: Boca Raton, FL

Paraskeva C and Hague A (1991) Colorectum. In *Human Cancer in Primary Culture*, John Masters JRW (ed), pp. 151–168. Kluwer Academic Publishers: Dordrecht, The Netherlands

Paraskeva C, Finerty S, Mountford RA and Powell SC (1989) Specific cytogenetic abnormalities in two new human colorectal adenoma-derived epithelial cell lines. *Cancer Res* **49**: 1282–1286

Pinto M, Robine-Leon S, Appay M-D, Kedinger M, Triadou N, Dussaulx E, Lacroix B, Simon-Assmann P, Haffen K, Fogh J and Zweibaum A (1983) Enterocyte-like differentiation and polarization of the human colon carcinoma cell line Caco-2 in culture. *Biol Cell* **47**: 323–330

Playford RJ, Hanby AM, Goodlad RA, Gschmeissner S, Patel K, Peiffer LP and McGarrity T (1995) The EGF-receptor is present on the basolateral, but not the apical surface of enterocytes in the human gastrointestinal tract. *Gastroenterology* **108**: A747

Radinsky R, Bucana CD, Ellis LM, Sanchez R, Clearly KR, Brigati DJ and Fidler IJ (1993) A rapid colorimetric in situ messenger RNA hybridization technique for analysis of epidermal growth factor receptor in paraffin-embedded surgical specimens of human colon carcinomas. *Cancer Res* **53**: 937–943

Radinsky R, Risin S, Fan D, Dong Z, Bielenberg D, Bucana CD and Fidler IJ (1995) Level and function of epidermal growth factor receptor predict the metastatic potential of human colon carcinoma cells. *Clin Cancer Res* **1**: 19–31

Rusch V, Baselga J, Cordon Cardo C, Orazem J, Zaman M, Hoda S, McIntosh J, Kurie J and Dmitrovsky E (1993) Differential expression of the EGFR and its ligands in primary non-small cell lung cancer and adjacent benign lung. *Cancer Res* **53**: 2379–2385

Stanton P, Richards S, Reeves J, Nikolic M, Edington K, Clark L, Robertson G, Souter D, Mitchell R and Hendler FJ (1994) EGFR expression by human squamous cell carcinoma of the head and neck, cell lines and xenografts. *Br J Cancer* **70**: 427–433

*© Cancer Research Campaign 1998*